# Supplementary material for: Improving the accuracy of automated labeling of specimen images datasets via a confidence-based process
Source: PLoS Comput Biol. 2025 Nov 12;21(11):e1013650. doi: 10.1371/journal.pcbi.1013650 (PMC12629457; doi:10.1371/journal.pcbi.1013650)
Supplement: Attachment — Submitted filename: PLOS Computational Biology Response to Reviewers-3.pdf [file pcbi.1013650.s001.pdf]

PLOS Computational Biology Response to Reviewers

We would like to thank the editor and reviewers for the time they devoted to provide feedback to improve our manuscript.

Below is the list of modifications we incorporated, based on the comments received.

Reviews

Editor Review

Comment 1

The results used to validate the effectiveness of the proposed mehtod were calculated on a dataset without the discarded sample. This makes the results questionable. The authors are encouraged to the effectiveness of the proposed method in general sence.

Response

Although we understand the concern of not using a higher rejection threshold for the “Flowering time shift” experiment, we believe that this experiment and the dataset shared alongside it is valuable to the community. It also acts as a counterpoint to the study replication example that required a high rejection threshold, allowing us to highlight the necessary precautions and methodology to examine how to choose a rejection threshold for a given task and dataset. Indeed, it shows that for certain tasks, although counter-intuitively in some cases, more data can be more statistically valid than better (but less) data. Lastly, by sharing the dataset associated with this experiment, we strongly believe that promising works can be enabled from within the community.

Comment 2

We note that the following section: Discussion is mentioned twice in the manuscript

Response

The outline has been fixed

Comment 3

Please upload a copy of Figures Fig. VII-A(a), and Fig. VII-A(b) which you refer to in your text on page 11. Or, if the figures are no longer to be included as part of the submission please remove all reference to them within the text. + Please ensure that all Figure files have corresponding citations

and legends within the manuscript. Currently, Figures 12, 13, and 15 in your submission file inventory do not have in-text citations. Please include the in-text citations of the figures.

Response

Broken internal references have been fixed.

Comment 4

Copyrighted materials and figure authorship

Response

All herbarium images have been checked and replaced in order to ensure they are all CC BY 4.0.

Composite figures have all been designed by hand by the authors.

Comment 5

Please number your tables with Arabic numerals and change the citations accordingly.

Response

Updated format

Comment 6

Please provide a completed 'Competing Interests' statement, including any COIs declared by your co-authors. If you have no competing interests to declare, please state "The authors have declared that no competing interests exist". Otherwise please declare all competing interests beginning with the statement "I have read the journal's policy and the authors of this manuscript have the following competing interests:"

Response

Statement added

Reviewer #1

Comment 1

The paper proposes a deep learning model confidence threshold framework that achieves arbitrarily high accuracy by sacrificing some samples. The accuracy reported here is calculated based on the discarded samples, which, in contrast to traditional accuracy computed across all classified samples, is essentially closer to precision. The authors should clarify the fundamental differences and advantages between the proposed approach and commonly used metrics, such as the ROC curve, which typically sacrifices recall to enhance precision (or vice versa).

Response

Added a paragraph discussing the difference between tuning an accuracy/recall tradeoff VS tuning an accuracy/rejection tradeoff and the practical similarities and differences.

Comment 2

Furthermore, the confidence output and prediction accuracy of deep neural networks can be effectively calibrated during training. Related techniques are discussed in the work of Moloud Abdar et al. in “A Review of Uncertainty Quantification in Deep Learning: Techniques, Applications, and Challenges”, which highlights numerous methods for achieving well-calibrated predictions. The authors are encouraged to consider incorporating calibration methods into the existing framework to enable more direct and accurate confidence threshold selection.

Response

Added a paragraph on Limitations and potential improvements.

One topic of this paragraph is the discussion about calibration and pointing to some additional works for readers that want to go further with it.

Although we understand the motivation to obtain models that are as good as possible, we believe that the impact of this work is stronger by keeping the model training as straightforward as possible, at the cost of some performance. Adding additional layers of complexity could give the impression that only models trained with such methods could benefit from the presented approach, which goes against our belief that the presented method is applicable and worthy even for models trained in a ‘traditional way’ by non-experts practitioners.

Comment 3

The experiments are conducted on fixed datasets without performing cross-validation. Are the reported metrics based on a single training run? The authors should provide further clarification on how they mitigate the potential for result variability.

Reponse

We added a clarification to the method description that we are indeed using a fixed dataset. Regarding using cross-validation, the authors believe that our training dataset is large and diverse

enough to be trained efficiently using a standard training/validation/test split, this being reinforced by the resulting model matching performances reported by similar studies training very similar models on similar datasets.

Comment 4:

Given that the results and discussion are already included in Section V, it is recommended that Section VI be renamed from "DISCUSSION" to "CONCLUSION".

Response

Outline has been fixed.

Comment 5

Fig. 15 requires revision as the data points are heavily overlapping. Additionally, the legend in subplot (a) is unclear.

Response

A cleaner legend has been added to the plot. Regarding the size of points, the authors believe that the points spread pattern is more important than the individual position of the points themselves. Having smaller points doesn't seem to improve that.

Comment 6

The header position in Table VI is inconsistent with other tables.

Response

Header has been fixed

Comment 7

Figure 16 also suffers from heavy overlap between points and lines

Response

In a similar vein as discussed in Comment 5, visualizing the noise in the spread of the points at various levels of thresholds (with various colors) is the most relevant information here, and smaller points make it harder to distinguish that in our opinion.

Comment 8

The "Definition" column in Table VII appears disorganized.

Response

Table formatting has been re-worked

Reviewer #2

Comment 1

Examples of rejected and accepted images
The authors illustrate in Fig. 15 the distribution of low-confidence images that are likely to be rejected by confidence threshold approach. However, this distribution is derived from ML layers, and they do not show actual images. The readers will be interested in whether the confidence level goes along well with human perception of ambiguous or clear flowering judgments. Therefore, the reviewer recommends adding example images for each confidence level (corresponding to green, yellow,…,black) and flowering/non-flowering.

Response

As suggested, examples of high-flowering, high non-flowering and low flowering confidence examples have been added and commented upon to provide some insight into the type of visual features that tend to correlate with these score.

Comment 2

Visualization for multi-class results
The reviewer recommends adding visualization similar to Fig. 15 for the multi-class results presented in Section V-C because the characteristics for the rejected images will be one of the main interests when using this approach.

Response

Although performing a thorough analysis of the multi-class performances of this particular model is appealing in itself, we believe that this section doesn't require further analysis to make the point that the proposed method applies and conserves the same properties in a multi-class setting compared to a 2-class setting.

Comment 3

The reviewer recommends providing a demo code for this approach so that readers can interact with the confidence thresholding effect.

Response

We appreciate this recommendation, but we are unable to generate such a product within the revision time frame.

Comment 4

Miscellaneous
On page 8, Table III, the reviewer believes the 2nd column in Nativity row should be native vs non-natives. Please check it.

The reviewer spotted a mislabeling of a figure. On page 11, Fig. VII should be Fig. 15. Please fix the mislabeling.

Response

Typos and mislabeling have been corrected

Reviewer #3

Comment 1

One major issue is that the method relies heavily on confidence scores produced by softmax layers. However, it is well-known that DNNs are often concerned as overconfident models. Thus, model calibration should be considered and discussed to enhance the reliability of the confidence-based method. Typical techniques include temperature scaling, isotonic regression, or calibration error metrics. I suggest the authors to consider improving the method with easy-to-implement calibration methods.

Response

Following a similar comment from another reviewer:

“Added a paragraph on Limitations and potential improvements.

One topic of this paragraph is the discussion about calibration and pointing to some additional works for readers that want to go further with it.

Although we understand the motivation to obtain models that are as good as possible, we believe that the impact of this work is stronger by keeping the model training as straightforward as possible, at the cost of some performance. Adding additional layers of complexity could give the impression that only models trained with such methods could benefit from the presented approach, which goes against our belief that the presented method is applicable and worthy even for models trained in a ‘traditional way’ by non-experts practitioners.”

Comment 2

Selecting only high-confidence examples may introduce sampling bias, as high-confidence samples may represent only those examples with simple visual patterns. The risk of sampling bias should be quantified or at least discussed, and potential mitigation strategies could be proposed.

Response

As with the calibration, we acknowledge that this point needed an additional discussion in the paper. We argue that this phenomenon is hard to mitigate, but the consequences can be easily recognized (high rejections of particular subsets, such as particular families or species) and mitigated through targeted human labeling or training of specialized models for harder sub-tasks.

Comment 3

The evaluation on the 600K dataset is not convincing enough since the subset used for evaluation covers only a small portion of species. If covering all the species is too expensive, you can at least consider a more reliable strategy for sampling a subset of representative examples (e.g., Core-Set) over the 600K dataset.

Response

While the evaluation subset included a small number of species, they represent many major evolutionary clades present in the 600K dataset and we feel they reflect the morphological variation present and thus do help to make a convincing case for the utility of the method.
