# Supplementary material for: Improving the accuracy of automated labeling of specimen images datasets via a confidence-based process
Source: PLoS Comput Biol. 2025 Nov 12;21(11):e1013650. doi: 10.1371/journal.pcbi.1013650 (PMC12629457; doi:10.1371/journal.pcbi.1013650)
Supplement: Attachment — Submitted filename: Response to Reviewers _2.pdf [file pcbi.1013650.s002.pdf]

PLOS Computational Biology Response to Reviewers

We would like to thank the editor and reviewers for the time they devoted to provide feedback to improve our manuscript.

Below is the list of modifications we incorporated, based on the comments received.

Reviews

Editor Review

Comment 1

Kindly revise your competing statement to align with the journal's style guidelines: 'The authors declare that there are no competing interests.'

Response

Associated sentence has been edited

Reviewer #1

-

Reviewer #2

Comment 1

Even if authors do not have time to make a full tutorial, I believe they should at least make their code used in the study open with minimal description for the reproducibility of the study.

Response

A code repository with two files are provided at the following url:
https://github.com/NouFuS/confident_based_deep_learning_for_ecology

It contains:

- an example of the accuracy/coverage figure generation process using a toy dataset.
- an example of usage of the 600k AI annotated dataset that reproduces the large-scale flowering shift analysis results presented in the paper.

Reviewer #3

Comment 1

Please add a small paragraph to discuss the potential risk of data bias by selecting high-confidence examples.

Response

A paragraph regarding that point has been added in the previous revision at position VII.7.b: 'Sampling Bias'
